# Flexible Transparent Heat Mirror for Thermal Applications

**DOI:** 10.3390/nano10122479

**Published:** 2020-12-10

**Authors:** Shimin Li, Qianqian Xu, Ziji Zhou, Wenchao Zhao, Xiaowen Li, Zhengji Wen, Yao Yao, Hao Xu, Huiyong Deng, Ning Dai, Jiaming Hao

**Affiliations:** 1 State Key Laboratory of Infrared Physics, Shanghai Institute of Technical Physics, Chinese Academy of Sciences, Shanghai 200083, China; lishimin@mail.sitp.ac.cn (S.L.); xuqianqian@mail.sitp.ac.cn (Q.X.); zhouziji@mail.sitp.ac.cn (Z.Z.); wczhao@mail.sitp.ac.cn (W.Z.); liwen_phy@163.com (X.L.); wenzhengji@mail.sitp.ac.cn (Z.W.); EoauYao@163.com (Y.Y.); haoxu@mail.sitp.ac.cn (H.X.); hydeng@mail.sitp.ac.cn (H.D.); 2 School of Electronic, Electrical and Communication Engineering, University of Chinese Academy of Sciences, Beijing 100049, China; 3 Hangzhou Institute for Advanced Study, University of Chinese Academy of Sciences, Hangzhou 310024, China

**Keywords:** Ag-doped ITO, transparent heat mirrors, thermal applications, flexible, transparent

## Abstract

Transparent heat mirrors have been attracting a great deal of interest in the last few decades due to their broad applications, which range from solar thermal convection to energy-saving. Here, we present a flexible Polyethylene terephthalate/Ag-doped Indium tin oxide/Polydimethylsiloxane (PAIP) thin film that exhibits high transmittance in visible range and low emissivity in the thermal infrared region. Experimental results show that the temperature of the sample can be as high as 108 °C, which is ~23 °C higher than that of a blackbody control sample under the same solar radiation. Without solar radiation, the temperature of the PAIP thin film is ~6 °C higher than that of ordinary fabric. The versatility of the large-area, low-radiation-loss, highly-transparent and flexible hydrophobic PAIP thin film suggest great potential for practical applications in thermal energy harvesting and manipulation.

## 1. Introduction

High efficiency and energy-saving technologies are highly desirable due to increasing global energy consumption [1,2]. Since most of the energy of objects is transferred or dissipated in the form of heat, thermal management is particularly important for reducing energy consumption and improving energy conversion [3,4,5]. Energy dissipation in the form of infrared (IR) radiation accounts for a very large proportion of heat dissipation. Therefore, the infrared emission properties of materials play vital roles in achieving efficient thermal management [6,7,8,9,10,11,12,13,14]. For the application scenarios which require transparency (such as greenhouse coverings and windows plates), thermal management becomes more difficult, because reducing the energy consumption of these cases put higher requirements on the optical properties of materials, namely, (a) low emissivity in the infrared range to reduce heat radiation dissipation and (b) high light transmittance in the visible range to ensure transparency.

**Figure 1:**
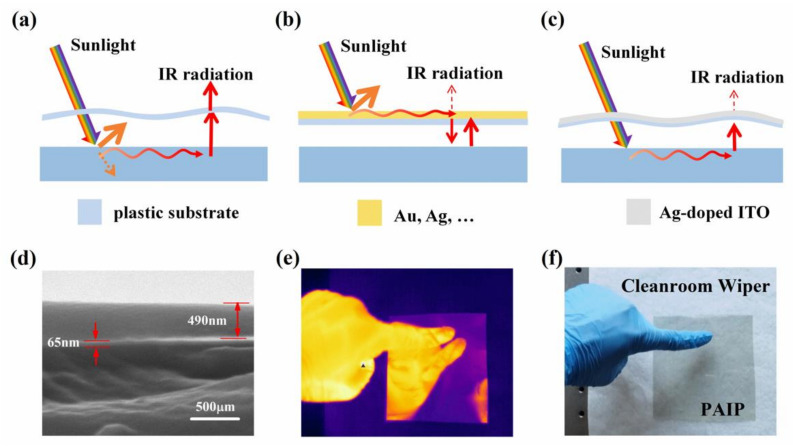
(**a**) Schematics illustration of object covered with normal transparent polymer thin film. (**b**) Object covered with metal. (**c**) Object covered with Ag-doped ITO thin film. (**d**) SEM photograph of the PET/Ag-doped ITO/PDMS (PAIP)sample’s cross section. (**e**) IR photography of PAIP sample to show the highly IR reflection. (**f**) Visible photography of sample to illustrate the transparency.

Despite the fact that conventional transparent materials, such as glass and organic thin films, have good transmittance in the visible range, the IR emissivity of these materials is very high and a large amount of energy will be dissipated by thermal radiation (Figure 1a). In addition, people have concentrated on developing novel metals as low IR emissivity materials [15,16,17]. A recently developed Ag film-coated textile has a low IR emissivity of about 4%, which can make the human body 7.1 °C higher than can be done by normal textiles. However, these materials have very low transmittance in the visible range and cannot be directly applied in greenhouses or windows (Figure 1b). In practice, transparent heat mirrors (THM) represented by transparent conductive oxide (Indium tin oxide (ITO), Aluminium doped Zinc Oxide (AZO), etc.) or dielectric/metal/dielectric multilayer systems (ITO/Ag/ITO, TiO_2_/Ag/TiO_2_, etc.) were investigated by many groups [18,19,20,21,22,23,24,25,26]. High transmittance (~80%) in visible range (400–800 nm) and extremely low emissivity (~10%) in IR range (3–20 um) were found. This unique optical property of THM shows great potential in thermal management of places that require light transmission [27,28]. Dopants such as Ti, Ge, and Ag to the ITO matrix were found to be helpful for enhancing both the electrical conductivity and optical transparency [29,30,31]. However, there have been only limited efforts and attempts to introduce them into applications of thermal management. Hence, we propose growing Ag-doped ITO on the flexible transparent substrate (polyethylene terephthalate, PET) to minimize the thermal loss of IR radiation (Figure 1c). Furthermore, the traditional conductive oxide is easily corroded by the acid in rain water, which weakens the infrared radiation characteristics and lifetime. Here, in order to avoid the direct contact with the rainwater, we applied an ultra-thin hydrophobic Polydimethylsiloxane (PDMS) to the surface of THM. This structure could not only prevent erosion of the acid rain, but also have the potential of self-cleaning.

In this study, by simultaneously engineering the optical property in solar as well as in long wavelength IR spectral regions, PET/Ag-doped ITO/PDMS (PAIP) thin films were designed. This structure can well meet the requirements on the optical properties of materials in thermal management applications for some specific environments mentioned above. Here, Ag-doped ITO were used for dropping the radiation. Complex refraction indexes of Ag-doped ITO were measured by spectroscopic ellipsometry. Furthermore, low heat loss performance is experimentally and numerically demonstrated. Experimental results show that our thin film can achieve appreciable heating effect of both indoor and outdoor environments.

## 2. Experimental Scheme

### 2.1. Synthesis of PDMS Solutions

Raw material of PDMS (SYLGARD184) with two components the pre-polymer base A (Dimethyl siloxane, dimethylivinyl terminated) and the cross-linking curing agent B (Dimethyl, methylhydrogen siloxane) were produced by Dow Corning Co (Shanghai, China). To get thin PDMS, the original PDMS solution was first diluted with toluene (Aladdin, Shanghai, China) in the ratio of 1:4 before adding B and then applying magnetic stirring for 60–80 min under room temperature. Subsequently, component B dropped into the solution. To reduce the curing time and make it being stiffer, the ratio of two components A and B is 5:1. This ratio is bigger than the normal ratio of 10:1 that manufacturers recommended and was finally fixed after attempts at different experimental conditions and analysis of reference [32]. Similarly, the whole mixture was stirred for 60–80 min under room temperature.

### 2.2. Sample Fabrication

**Figure A1:**
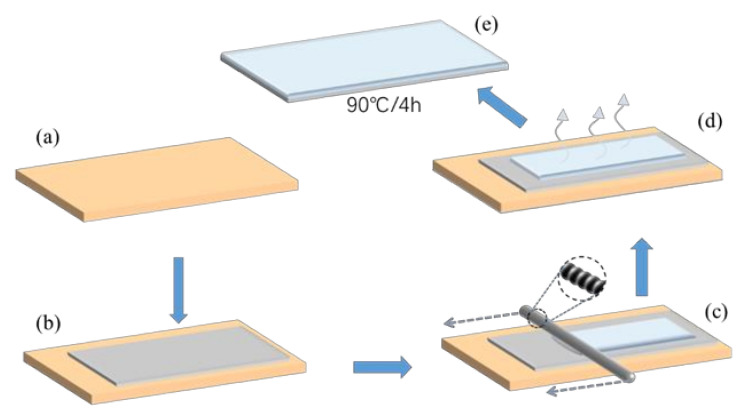
Flow chart of PAIP preparation. (**a**) Clean the crap-board. (**b**) Lay the PAI film flat on the surface of the crap-board. (**c**) Scraping PDMS solution onto PAI. (**d**) Remove the solvent of PDMS. (**e**) Curing the PDMS.

The PET/Ag-doped ITO thin film was bought from South China Science & Technology Co., Ltd. (Shenzhen, China). The PDMS was deposited onto the PET/Ag-doped ITO film by the flat membrane wiping equipment (BEVS-1811/2, BEVS industrial co., Ltd., Guangzhou, China). The wiping thickness of PDMS was 4 μm and the speed was 10 mm/s. Then, the sample was heated in a drying box at 100 °C for 240 min. The preparation steps are illustrated in Figure A1 (Appendix A).

### 2.3. Optical Characterization

For the Ultraviolet-Visible-Near Infrared (UV-VIS-NIR) part, the room temperature reflectance spectra of the specimen was measured by an Agilent Cary 7000 (Penang, Malaysia) spectrophotometer with a resolution bandwidth of 2 nm and the incident light was almost perpendicular to the sample surface (6°). For the Mid-infrared (MIR) part, transmission and reflection of these thin films were characterized by using a Micro-Fourier Transform infrared (Micro-FTIR), (Thermo Scientific, Nicolet iN10, Waltham, MA USA). A gold film was used as a reference and the incident angle of light source was 30°.

### 2.4. Thermal Measurement

The temperature of the sample was detected by a T type thermocouple and a multichannel data acquisition unit (TOPRIE, TP700, Shenzhen, China) was utilized to collect and store the data under the rate of 1 time per second. In the indoor experiment, a high-density polyethylene foam board (50 mm × 50 mm × 20 mm) was developed as the sample holder. The thin film sample was pasted on the outer surface of the holder. The other outer surfaces of the foam board were covered by aluminum foil to decrease heat dissipation on foam by IR radiation. In the outdoor experiment, a high-density polyethylene foam board (90 mm × 90 mm × 20 mm) with an inverse pyramid type was developed as the sample holder. This kind of holder ensures that only the top side was directly exposed to the sunlight. The thin film sample was directly pasted on the top side of the foam board and the other sides the foam board were covered by aluminum foil.

### 2.5. Numerical Simulations

Numerical simulations were conducted based on the finite-difference-time-domain (FDTD) method [33], to investigate the IR reflection (*R*) and absorption of the sample. In the simulations, the source with the wavelength range of 2.5–20 µm was launched into the two-dimension FDTD simulation zone. Periodic boundary conditions were imposed along the x axes. 1 nm finer meshes were adopted.

## 3. Results and Discussions

### 3.1. Morphological and Structural Characteristics

**Figure A2:**
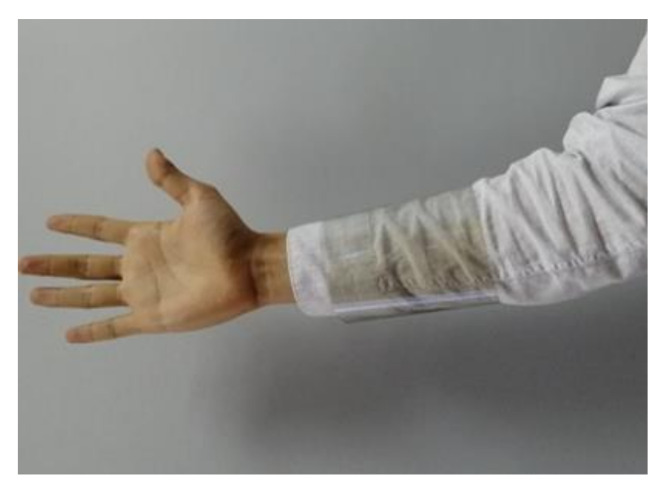
Illustration of the flexibility of the PAIP sample.

**Figure A3:**
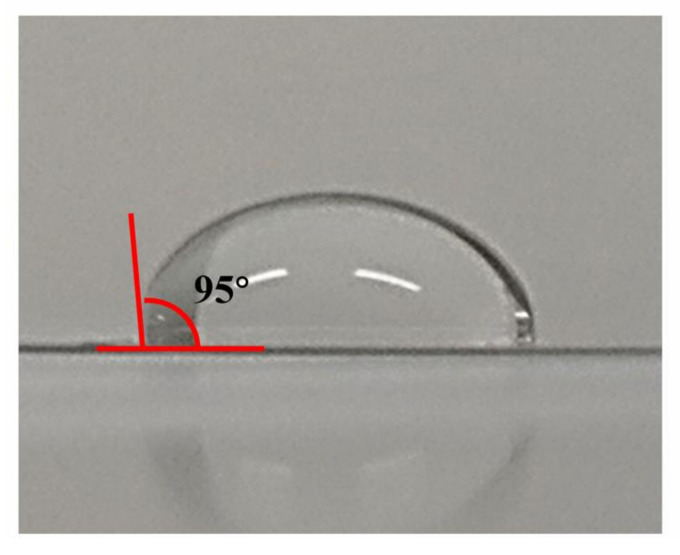
Photograph of the contact angle *θ*.

To investigate the microstructure of ITO thin film, we take the SEM image of section of PET/Ag-doped ITO/PDMS thin film (shown in Figure 1d). The transparent (~170 μm in thickness) PET thin film with good flexibility was adopted as the substrate. The low-emissivity and hydrophobic coating consists of a thin layer of Ag-doped ITO (65 nm in thickness) and an optically thick PDMS layer (~490 nm in thickness). The hydrophobic thin PDMS layer can not only play an important role in improving the corrosion resistance of thin film, but also provide a self-cleaning function (the contact angle was shown in Figure A3 (Appendix A)). As a result, the unique three-layer design presents three major functionalities: (1) a self-cleaning function with hydrophobic thin PDMS layer, (2) low MIR radiation because of the highly reflective Ag-doped ITO coating, and (3) high transparency in sun light. To illustrate the flexible nature of the proposed material, a photograph of this sample on an arm is presented in Figure A2.

### 3.2. Optical Properties

**Figure 2:**
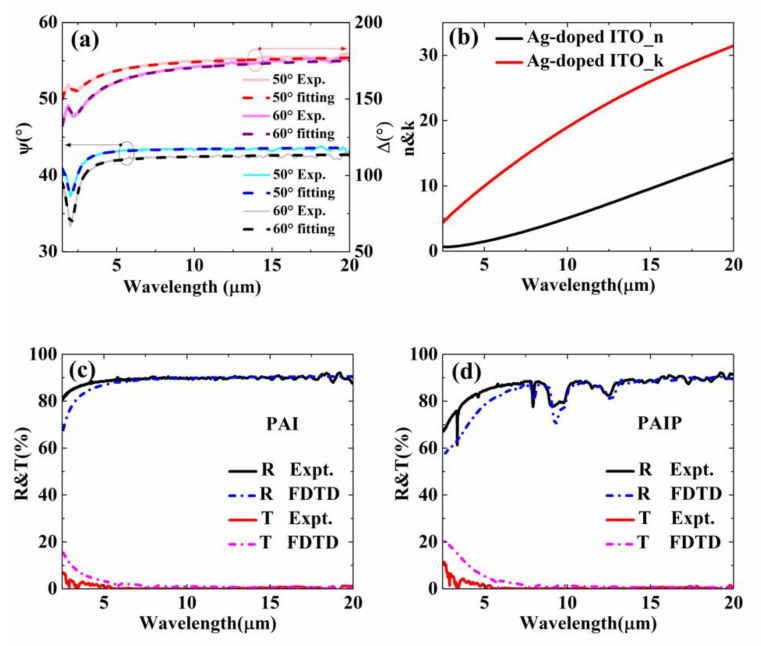
(**a**) Ellipsometry data Ψ and Δ (solid curves) for a 65 nm thick Ag-doped ITO thin film in the spectral range of 3–20 μm at incident angles θ = 50°, 60°. (**b**) Optical constant of Ag-doped ITO. (**c**) Reflection and transmission spectrum of the PAI sample. (**d**) Reflection and transmission spectrum of the PAIP sample.

**Figure A4:**
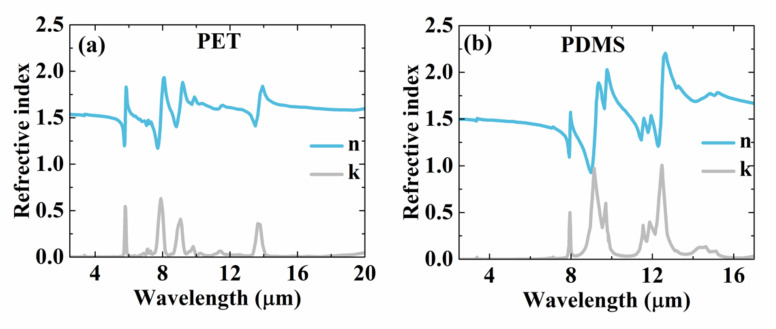
Optical constant of different materials. (**a**) PET and (**b**) PDMS in IR range [34,35].

**Figure A5:**
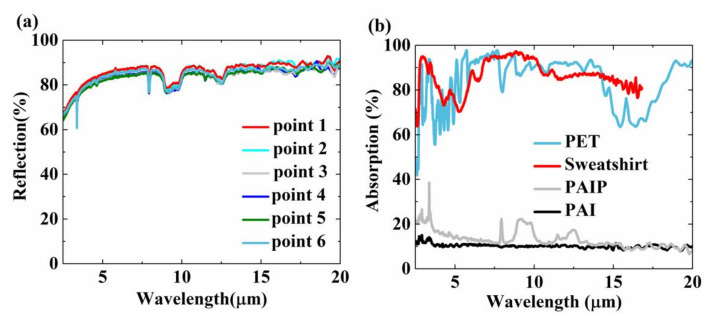
(**a**) Reflection spectrum of different points of the PAIP sample. (**b**) Absorption of four kinds of different materials.

Long wavelength infrared properties were first investigated because they directly impact thermal radiative loss. Figure 2a shows the measured ellipsometry data Ψ and Δ (solid curves) for a 65 nm thick Ag-doped ITO thin film in the spectral range of 3–20 μm at incident angles *θ* = 50°, 60°, respectively. The Ag-doped ITO’s complex refractive index components n and k were extracted by model fitting experimental data obtained from spectroscopic ellipsometry (see Figure 2b). The refractive indexes of PET and PDMS are taken from reference and were plotted in Figure A4 [34,35]. As one can see, the overall trend of fitting results is consistent with the same results experimentally measured. To investigate the optical performance of the multilayer structure insight, we also performed numerical simulations with the same geometric parameters. The simulations are conducted by using Finite-Difference-Time-Domain (FDTD) methods. Figure 2c,d displays the reflection and transmission of PET/Ag-doped ITO (PAI) and PAIP thin film. Nearly 90% of IR light reflection was found by PAI sample. This high reflection can be attributed to the layer of Ag-doped ITO. Compared with PAI thin film, no significant change has been found in PAIP in the range of 5–20 μm except a limited drop in 10 and 12.57 μm. Note that the transmission for the two structures was almost zero (see dot line). Therefore, we can denote that the absorption of the two structures is about 10%, using the formula of *A + R + T* = 1 (*A* denotes absorption; *R* denotes reflection; *T* denotes transmission). To identify the uniformity of the PDMS layer, six different points were chosen to measure the reflection (Figure A5a). The result shows the good homogeneity of our sample. Given that the power of thermal radiation is determined by the IR absorption (or emission) of materials, we measured the absorption of the two other samples (sweatshirt and PET). As the result shows in Figure A5b, the absorption/emission of samples of PAI and PAIP was only ~10%, which ensures less energy loss than normal materials through IR radiation.

**Figure 3:**
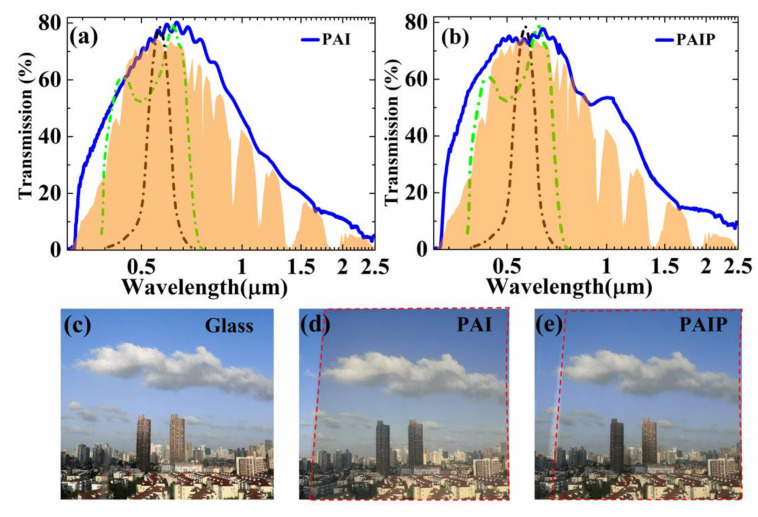
Solar range spectrum of samples. (**a**) Transmission spectrum of the PAI sample. (**b**) Transmission spectrum of the PAIP sample. The green line represents the plant response spectrum, the black line represents the human eye response spectrum, and the yellow area represents the normalized AM1.5 solar spectrum. (**c**) Transparency illustration of glass. (**d**,**e**) Transparency illustration of PAI and PAIP sample under the same surroundings (area in red dot line).

To further illustrate the optical properties of our samples in the solar range, we also measured the transmission in 0.3–2.5 μm range (Figure 3a,b). As we can see in Figure 3a, the transmission of PAI reaches its maximum at 660nm, which corresponds to the peak of the plant response [36]. Similar characteristics in transmission were found in PAIP (Figure 3b) and differences appeared in the small widening of spectroscopic curve around visible range. This phenomenon may be attributed to the rising of refractive index gradient when the refractive index of PDMS is between the air and the Ag-doped ITO layer in the solar spectrum. More than ~73% solar energy can pass though the PAIP thin film due to its good transparency around the visible region (0.4–1.0 μm). With the introduction of the PDMS thin layer, the absorption dropped down around 10%. Figure 3c shows a photograph of the view outside a normal window glass. Figure 3d,e shows PAI and PAIP film applied to an actual window to assess the effect on visibility. The clearly visible exterior proves the high visible light transmittance of PAIP film.

### 3.3. Heating Performance

**Figure 4:**
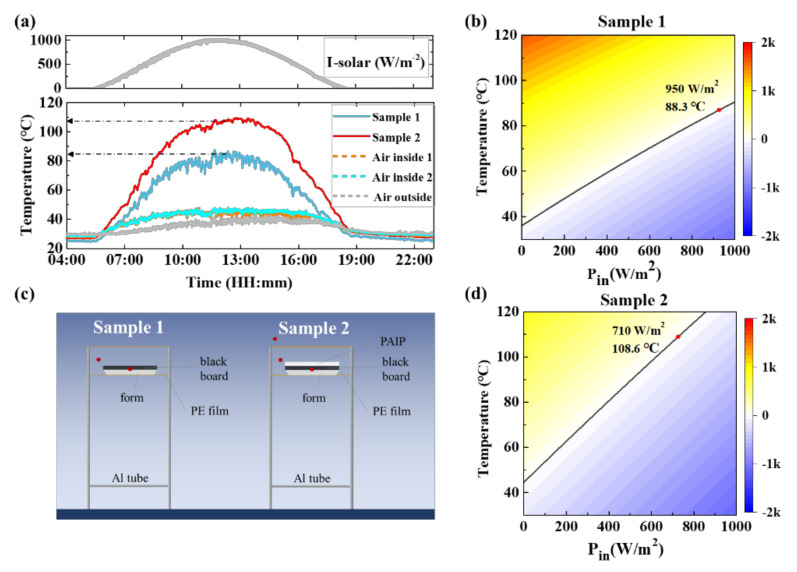
Outdoor test results. (**a**) Solar radiation intensity and temperature of samples. (**b**) Temperature simulation result of sample 1 under different input power. (**c**) Device setup of outdoor heat conversion test. (**d**) Temperature simulation result of sample 2 under different input power.

**Figure A6:**
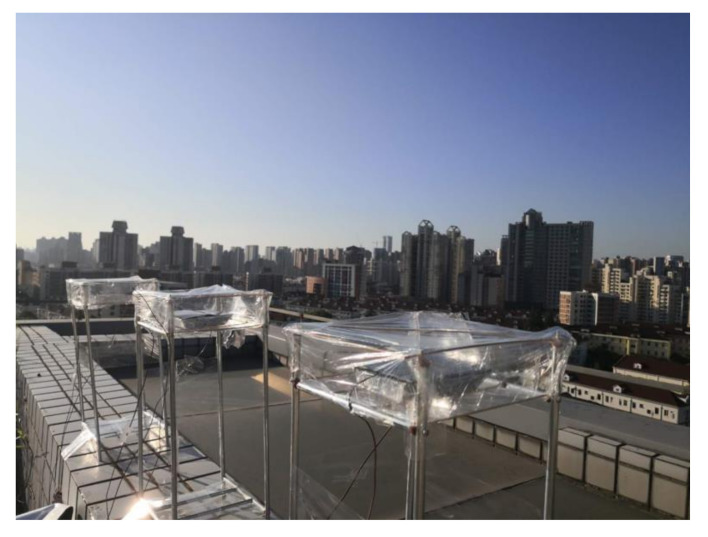
Photography of outdoor test.

In order to experimentally demonstrate the heating effect of our sample, all-day continuous tests were performed under a peak solar intensity *I*_solar_ of ~1000 W/m^2^ on a clear summer day in Shanghai, China (31° N, 121° W, 18 m altitude) (Figure A6). The sample was placed on a foam board that covers an Al foil at the lower surface to prevent heat loss from below. The frame structure wrapped by PE film was constructed as the cavity to prevent air flow convection (Figure 4b). Black polymer thin film (Sample 1) with high absorption in both solar and long IR range (thickness ~500 um) was covered on the upside of the foam board to absorb the input solar energy. In contrast, black polymer thin film covered by PAIP (Sample 2) was put on the foam board to show the radiative heating effect. Figure 4a shows the solar radiation from 4 am to 10 pm and how the pick of radiation intensity reached ~1000 W/m^2^. All temperature data were detected and recorded every five seconds by thermal coupling with precision of 0.01 °C. Before testing, we put all the channels of the detectors in the same place to check whether all of them are equal, so we could make sure that the difference of temperature between each channel in each test only depends on the actual temperature. The smooth result of solar radiation indicates that the sky was very clear and without any clouds. The temperatures of different points in the same time range were also shown in Figure 4a. Under the effect of sun light radiation and thermal conduction from the sample, the temperature of the air inside the cavity also reached ~47 °C, which was 8 °C higher than the outside air. The temperature of sample 1(blue line) and sample 2 (red line) were up to ~85 °C and ~108 °C at noon, respectively. We hold that the low emission of IR radiation is the reason why the temperature of sample 2 higher than that of sample 1. This phenomenon identifies the feasibility in real applications in outdoor heating for building, transportation facility, or the human body.

**Figure 5:**
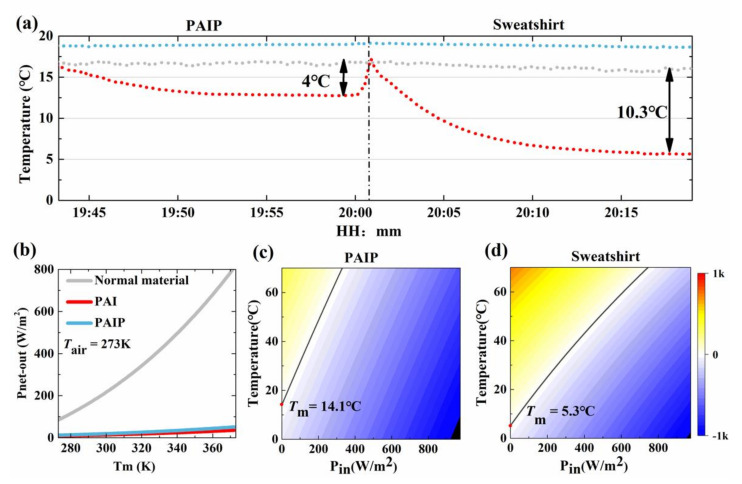
Indoor test results. (**a**) Experimental results of three points. (**b**) Intensity of *P*_net-out_ of different materials under different temperature. (**c**) Temperature simulation result of indoor test of PAIP. (**d**) Temperature simulation result of indoor test of Sweatshirt.

**Figure A7:**
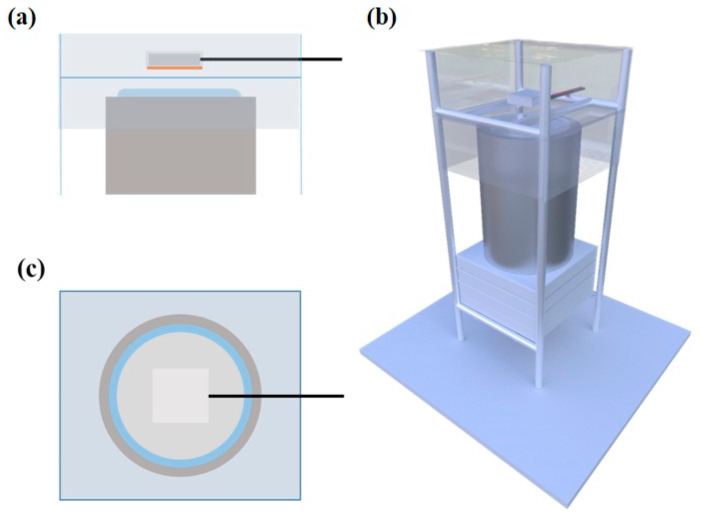
Illustration of indoor test.

To evaluate the impact of PAIP on personal heating in indoor environments when there was no solar radiation, indoor experiments were also identified [37]. A white sweatshirt was used as the reference to show the passive heating efficiency of PAIP. The schematic diagram of the indoor experimental test is shown in Figure A7. The temperature curve of three points (air at the lower surface of the cavity, air at the upper surface of cavity, and lower side of the sample) were measured and the results were shown in Figure 5a. Only a 4 °C temperature difference (Δ*T*) was found under PAIP coverage. As we can see, the Δ*T* of the PAIP sample was smaller than that of the sweatshirt by ~6.3 °C. This indicates that the PAIP sample has a better localized heating ability than normal cloth fabric and can minimize the heat loss without solar radiation.

### 3.4. Heating Performance Simulation

To provide further theoretical insight, radiative heating calculations were performed to reveal the heating behavior of our thin films. Taking non-radiation conduction (*P_nonrad_*) and other input energy source (*P_in_*) effects into account, we simulated the net out-put power *P_net_* under different *T*_m_ [38,39].

(1) $$P_{net}=P_{r}-P_{a}+P_{nonrad}-P_{in}$$

Here, *P_r_* is the radiative power emitted by the structure and *P_a_* is the amount of the incident thermal radiation absorbed by the surface from the atmosphere [38,39].

(2) $$P_{a}=2\pi \int_{0}^{\pi /2}\sin\theta \cos\theta d\theta \int_{0}^{\infty }U_{B}\left(T_{air},\lambda \right)\varepsilon_{r}\left(\lambda ,\theta \right)\varepsilon_{a}\left(\lambda ,\theta \right)d\lambda$$

(3) $$P_{r}=2\pi \int_{0}^{\pi /2}\sin\theta \cos\theta d\theta \int_{0}^{\infty }U_{B}\left(T_{air},\lambda \right)\varepsilon_{r}\left(\lambda ,\theta \right)d\lambda$$

where $U_{B}\left(T,\lambda \right)=\frac{2hc^{2}}{\lambda^{5}}\frac{1}{e^{hc/\lambda k_{B}T}-1}$, is the spectral radiance of a black body defined by Planck’s law at any temperature *T*, where *h* is Planck’s constant, *k_B_* is the Boltzmann constant, *c* is the speed of light in vacuum, and *λ* is the wavelength. *T_m_* and *T_air_* denote the temperature of the atmosphere and object. To simplify the numerical calculation, angle differences were neglected and *ε_r_* (*λ,θ*) and *ε_a_* (*λ,θ*) were treated to be *ε_r_* (*λ*) and *ε_a_* (*λ*). The emissivity *ε*_r_ (*λ*) of the radiator can be defined by its absorptivity *ε  = α =  *1 *– ρ − τ* (*ε*, *α*, *ρ*, and *τ* are emissivity, absorptivity, reflectivity, and transmissivity, respectively) according to Kirchhoff’s law. The amount of *P_nonrad_* can be calculated as *P_nonrad_* = *h*(*T_a_ − T_r_*), where *h* (*h* = *q*_cond_ + *q*_conv_) is the heat exchange coefficient combining contributions from conduction and convection. During simulation, we understood that the experiment result, the same air temperature (47 °C) was set in the calculation. When the system is in thermal equilibrium, *P_net_* will be zero.

Figure 4b,d shows the result of PAIP and normal material. The color map represents the value of *P_net_* and black line mean *P_net_* = 0 W/m^2^. As we can see in Figure 4b, the thermal equilibrium temperature increased linearly by increasing Pin. Comparing Figure 4b,d, sample 2 (black board covered PAIP) shows a bigger slope than the uncovered one. When *P_in_* reaches 706 W/m^2^, the equilibrium temperature can be 108.6 °C, which is higher than the surrounding environmental temperature (*T_air_*) of 61.6 °C. In contrast, sample 1 (normal black material) can only reach 88.3 °C. It is noted that PAIP film can achieve a higher temperature than normal materials under the same sun light situation. The values are consistent with the experiment results, and indicate PAIP’s potential to reduce the energy loss of greenhouse or buildings. Similarly, indoor simulation was performed and the result shown in Figure 5b,c. The steady state temperature of PAIP sample and sweatshirt is 14.1 °C and 5.3 °C, respectively. Good agreements are noted between the numerical and experimental results (less than 1 °C difference).

**Figure A8:**
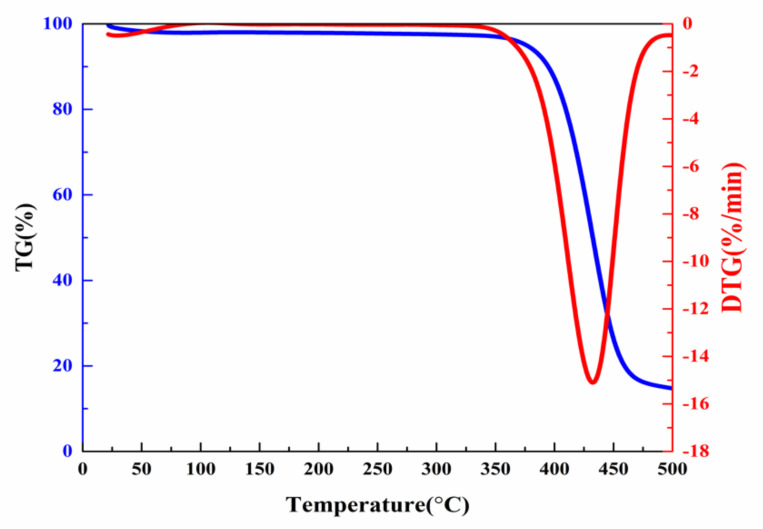
The thermal stability of the PAIP. The blue line corresponds to mass loss ratio of the PAIP sample and the red line corresponds to derivatives of mass loss ratio of the PAIP sample.

To further understand the reason why PAIP sample has higher temperature under the same conditions, we also calculated the relationship between the temperature of material (*T*_m_) and net output radiation power (*P*_net-out_). Figure 5b shows the net radiative loss power *P_r_* − *P_a_* of different materials under different temperature. The ambient temperature was set as 273 K. As we can see, with the increase of the temperature of normal high emission materials (cotton fabric, polymer, carbon, etc.), the loss of Pnet-out increases dramatically. When the material’s temperature reaches 370 K (97 °C), the radiative loss can be as high as 800 W/m^2^. And even at a temperature close to that of human bodies 310 K (37 °C), there is also 282 W/m^2^ energy being lost through the radiative exchange, 4 times bigger than normal human body heat dissipation power (~75 W/m^2^). In contrast, low emission materials (PAI or PAIP thin film) reveal loss power smaller than 50 W/m^2^ and very little shift of intensity. It indicates that the capacity of the heat dissipation of PAIP film was much lower than normal material. To illustrate the thermal stability of the proposed material, the thermal stability test result of our sample is presented in Figure A8. The sample is relatively stable before 350 °C. The decomposition reaction of PET substrate begins at about 350 °C and completes at about 480 °C. The mass loss of thermal decomposition is 84.67%. The maximum decomposition rate of the sample is at 433.5 °C.

## 4. Conclusions

In summary, we have proposed and demonstrated flexible transparent PET/Ag-doped ITO/PDMS thin films. Lower IR emissivity of Ag-doped ITO suppress the thermal loss through IR radiation under indoor and outdoor environment. Highly transparency hydrophobic PDMS surface makes sure it can be directly applied in greenhouses or windows without the fear that there will be erosion caused by acid rain. When the blackboard is covered with the PAIP, the temperature can reach 108 °C, which is 23 °C higher than the temperature without PAIP film under the same external environment. The indoor experiment also shows that PAIP film can reach a temperature difference of 6 °C, which is far better than that of ordinary fabric materials. The heat transfer model was adopted to understand our experiment results, which considered heat loss (radiative loss, convective/conductive heat loss) as well as other input power. The combination of soft substrate, high visible transparency, broad infrared low emission, and hydrophobic surface demonstrated in our study shows great potential in real high-performance applications in energy-saving systems.
